# Progress and challenges in the use of fluorescence‐based flow cytometric assays for anti‐malarial drug susceptibility tests

**DOI:** 10.1186/s12936-021-03591-8

**Published:** 2021-01-21

**Authors:** Kasem Kulkeaw

**Affiliations:** Department of Parasitology, Faculty of Medicine Siriraj Hospital, Mahidol University, 2, Wanglang Road, Bangkoknoi, 10700 Bangkok, Thailand

**Keywords:** Malaria, Flow cytometry, *Plasmodium falciparum*, Fluorochrome

## Abstract

Drug-resistant *Plasmodium* is a frequent global threat in malaria eradication programmes, highlighting the need for new anti-malarial drugs and efficient detection of treatment failure. *Plasmodium falciparum* culture is essential in drug discovery and resistance surveillance. Microscopy of Giemsa-stained erythrocytes is common for determining anti-malarial effects on the intraerythrocytic development of cultured *Plasmodium* parasites. Giemsa-based microscopy use is conventional but laborious, and its accuracy depends largely on examiner skill. Given the availability of nucleic acid-binding fluorescent dyes and advances in flow cytometry, the use of various fluorochromes has been frequently attempted for the enumeration of parasitaemia and discrimination of *P. falciparum* growth in drug susceptibility assays. However, fluorochromes do not meet the requirements of being fast, simple, reliable and sensitive. Thus, this review revisits the utility of fluorochromes, notes previously reported hindrances, and highlights the challenges and opportunities for using fluorochromes in flow cytometer-based drug susceptibility tests. It aims to improve drug discovery and support a resistance surveillance system, an essential feature in combatting malaria.

## Background

Malaria caused by the parasitic protozoan *Plasmodium falciparum* is highly virulent [1] and a leading cause of mortality in children compared to other *Plasmodium* species. Given the occurrence of anti-malarial drug resistance [1, 2], a surveillance system to restrict spreading across endemic areas and new drug development are necessary to cope with ongoing global threats. Both procedures primarily rely on conventional culture of *P. falciparum*, either laboratory strains or field isolates, and microscopic examination to assess the growth of the cultured *Plasmodium* parasites [3–6]. Despite its standardization and conventional use, microscopic assays to enumerate and differentiate various stages of intraerythrocytic *Plasmodium* parasites are tedious and time-consuming. Moreover, interrater variability among the microscopists largely affects the interpretation, emphasizing a need for rapider and simpler assays that retain accuracy.

**Fig. 1:**
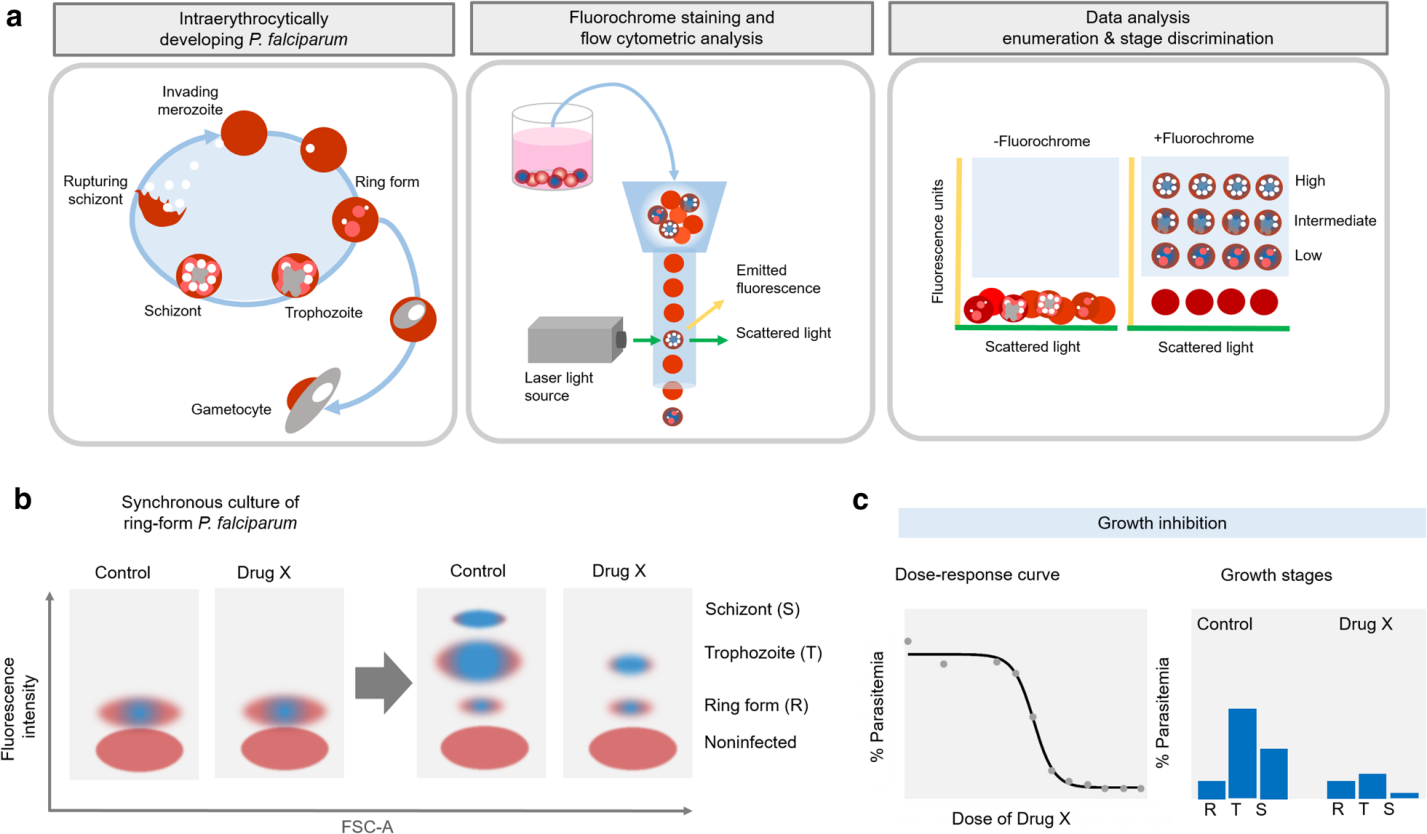
Use of a fluorochrome-based flow cytometer assay for the assessment of *Plasmodium falciparum* development. **a** Schematic diagram of the process to assess *Plasmodium* development in a culture using fluorescence dyes and flow cytometry. In the left panel, sexual and asexual development of *P. falciparum* are shown. *Plasmodium* parasites asexually develop through the following stages in a cyclical manner in culture: ring-shaped trophozoites, trophozoites, and schizonts. Upon rupture of a schizont, merozoites egress from the host cells and subsequently invade new erythrocytes, initiating the next developmental cycle. Some ring-shaped trophozoites undergo sexual development, resulting in gametocytes. The middle panel shows that to detect *Plasmodium*-infected erythrocytes, parasite nucleic acids of all intraerythrocytic stages could be stained with a number of fluorescent dyes. Then, the cells are taken into a flow cytometer, by which each cell is individually treated in a flowing fluid. The cells are exposed to light of different wavelengths generated from the laser light source. Nucleic acid-binding fluorochromes can be excited by light, resulting in fluorescence emission. In addition, cell size and intracellular content also cause scatter of the exposed light in the forward and side directions. A flow cytometer obtains a fluorescence signal from the emitted fluorescence and the forward- and side-scattered light. The fluorescence signal is displayed based on the fluorescence intensity. The emitted fluorescence is shown as a yellow Y-axis, while the forward scatter (FSC) or side scatter (SSC) is shown as a green X-axis (right panel). In the right panel, an example of data analysis post flow cytometry is illustrated. To determine the threshold to distinguish cells with emitted fluorescence, an unstained cell sample (− fluorochrome) is included. For the stained cells (+ fluorochrome), a mixture of cells is clearly discriminated based on resolving the emitted fluorescence intensity into three categories: high, intermediate and low. Here, when the fluorescence intensity is proportional to the nucleic acid content, each developmental stage of *Plasmodium* is identified: ring-form and early trophozoites have the lowest fluorescence intensity, late schizonts have intermediate fluorescence intensity, and mature schizonts have the highest fluorescence intensity. **b** An application of fluorochrome-based flow cytometric analysis for drug susceptibility testing. Generally, a synchronous culture of ring-formed *P. falciparum* is incubated with an anti-malarial drug (here referred to as X) and then stained with fluorochrome. Without drug X (control), the ring-form stage of *P. falciparum* develops into trophozoites and schizonts. In contrast, the drug-treated *Plasmodium* parasites develop at slower speeds or arrest between the ring form and the mature trophozoite, resulting in a lower percentage of trophozoites. **c** Examples of analysed data displayed in two formats. First, a dose-response curve can be generated regardless of the *Plasmodium* stage, but only parasitaemia, the percentage of cells having a fluorescence signal, is calculated. Second, according to the developmental stage, the bar graph can additionally inform which stage is affected by the drug. Based on the displayed data, drug X likely inhibits the development of the ring form into mature trophozoites

Flow cytometry, a laser-based measurement of cell characteristics, allows us to analyse a greater number of cells in a short period of time. In addition to measuring cell size and intracellular contents based on scattered light (Fig. 1), advances in fluorescent dye-labelled antibodies have increased the applicability of flow cytometry to distinguish various cell types in complex biological samples. Flow cytometry also facilitates quantitative analysis of rarely observed cells owing to the rapid liquid flow and high sensitivity of laser-excited fluorescence detection. Given that mature erythrocytes, host cells of blood-dwelling *Plasmodium*, lack nuclear DNA, detection of malarial DNA is thus able to distinguish parasitized erythrocytes from non-infected erythrocytes, especially those in a leukocyte-free culture of *Plasmodium* parasites. At present, many DNA- and RNA-binding fluorochromes are commercially available, and they are divided into two types: cell membrane permeable and non-permeable (Table 1). As a supportive but not alternative tool to conventional microscopy, the use of these fluorochromes enables anti-malarial drug testing in a fully or partially high-throughput setting. Therefore, in the first part of the review, methods used for assessing drug susceptibility tests and their limitations are summarized. Then, the use of flow cytometry in the measurement of parasitized erythrocytes is revisited. The advantages and remaining challenges of using nucleic acid-binding fluorochromes are highlighted. This review notes opportunities to overcome the limitations associated with each fluorochrome.

**Table 1:** Comparison of fluorochromes used in flow cytometric analysis of *Plasmodium* development and drug susceptibility tests

Fluorochrome (Maximum excitation/emission)	*Plasmodium species*	Process		Characteristics of assay			Applications		References
		Combination with RNA-binding fluorochrome	Cell fixation	% Lowest parasitemia	Staging intraerythrocytic development	Gametocyte detection	Merozoite invasion assay	Antimalarial drug assay	
*Cell membrane permeable*									
Hoechst 33,258 (350/461 nm)	*Falciparum*	None	Yes	0.001% (estimated from number of leukocyte)	No	ND	ND	ND	[35]
Acridine orange	*Falciparum*	None	Yes	Comparable to microscopic examination	Yes	ND	ND	Yes	[54]
DNA: 500/526									
RNA: 460/650									
Hoechst 33,342 (350/461 nm)	*Falciparum* *vivax & berghei*	Dihydroethidium	No	0.02%	Yes	ND	ND	Yes	[44]
ViSafe Green (488/520)	*Falciparum*	None	No	0.001%	Yes	Yes	ND	Yes	[61]
*Cell membrane impermeable*									
Propidium iodide (351/617 nm)	*Falciparum*	None	Yes	7.5%	Yes	ND	Yes	Yes	[62, 63]
PicoGreen		None	Yes	0.1%	ND	ND	ND	Yes	[75]
SYBR Green I (497/520 nm)		None	Yes	ND	Yes	ND	Yes	Yes	[53]
YOYO-1 (488/520–530 nm)		None	Yes	ND	Yes	ND	ND	Yes	[73]

### Role of light microscopy and ***in vitro*** assays in anti-malarial drug susceptibility tests

The use of a light microscope paved the way to discovering many pathogenic microorganisms causing human diseases, advancing our knowledge of medicine. Without staining with a dye, Alphonse Laveran first noticed an unknown microorganism with actively mobile filaments in blood taken from an infected soldier under a microscope, later confirming it as a *Plasmodium* male gamete with moving flagella [7]. Once the Giemsa solution, a type of Romanowsky dye, was deployed to stain human blood smears on glass slides, scientists were able to identify *Plasmodium*-infected erythrocytes under a light microscope. Thus, enumerating the parasitized erythrocytes and discriminating intraerythrocytic stages allows an estimation of the number of infected host cells (parasitaemia) and a measurement of parasite growth. Since then, Giemsa-based microscopy has become the gold standard for the diagnosis and clinical management of malaria [8] and an essential part of anti-malarial drug susceptibility tests.

To assess the effect of a drug on the intraerythrocytic growth of malaria parasites, cultures of *Plasmodium* spp. have been widely used as a malaria model in the preclinical phase of anti-malarial drug development and as a tool for drug resistance surveillance. To model malaria *in vitro*, laboratory-adapted *P. falciparum* strains or field-isolated strains are cultured with human erythrocytes, mostly those of blood group O, in RPMI 1640 medium supplemented with HEPES, sodium bicarbonate, and heat-inactivated human AB serum. The *in vitro* or *ex vivo* cultured *Plasmodium* parasites are then incubated with serial dilutions of drug and subjected to enumerating parasitized erythrocytes (schizonts) using a microscope. Giemsa-based light microscopy is the main method used to measure the intraerythrocytic development of *Plasmodium* parasites [9, 10], and it has a limit of detection of 0.001% parasitaemia based on examination of thick blood film in routine microscopic diagnosis [11]. In *Plasmodium* growth inhibition assays, a synchronous culture is treated with a drug [12]. Then, the development of *Plasmodium* parasites is examined. There are several methods for the assessment of intraerythrocytic *Plasmodium* growth: morphological observation under a microscope and biochemical measurements. For morphology-based assays, the microtechnique developed by Rieckmann et al. [3] is simple and reliable and has still been used in recent reports [13–18]. For biochemical assays, the microtechnique was modified to a semiautomatic tritiated hypoxanthine incorporation assay, measuring *Plasmodium* uptake of radiolabelled hypoxanthine, a nucleic acid precursor, to assess the growth inhibitory effect of anti-malarial drugs [19] or immune serum [20]. Given a rapid, more precise and quantitative method, many recent reports have been adopted for the assessment of *Plasmodium* growth inhibition in experiments [21] and in a clinical trial setting [22] and for surveillance of drug resistance in field isolates [23]. In addition to DNA synthesis-based assays, *Plasmodium* parasites in intraerythrocytic stages also actively synthesize cell membranes composed of phospholipids; thus, an assay based on incorporation of the radioisotope-labeled phospholipid precursor ethanolamine was developed [24], allowing an assessment of anti-malarial compounds targeting enzyme functioning in fatty acid biosynthesis [25]. Apart from biosynthesis-based tests, measurements of *Plasmodium*-specific lactate dehydrogenase [26–28] and histidine-rich protein II [29, 30] are also available and have been widely used for anti-malarial drug tests [31, 32].

In 1979, Howard et al. [33] were the first to use the nucleic acid-binding fluorescent Hoechst 33,258 dye to detect *P. berghei*-infected mouse erythrocytes. In early 1990, a fluorescence-based flow cytometer was introduced for drug susceptibility testing and parasite detection in human blood [34, 35]. Compared to the gold standard Giemsa-based microscope, fluorescence-based flow cytometry consumes a relatively shorter period of time. Prior to microscopic examination, thick blood film preparation and Giemsa staining were required. Then, a well-trained microscopist enumerates *Plasmodium*-infected cells and leukocytes. Approximately 16–20 h of drug testing was performed in a 96-well plate, while fluorescence-based flow cytometry consisting of cell staining, washing and acquisition was performed within 2 h [36]. Despite its high sensitivity, reliability and applicability for high-throughput experiments, fluorescence-based flow cytometry is expensive. Large, complex flow cytometers have been transformed into a small, transportable, user friendly and low-cost format, providing a platform suitable for field settings [36–38]. Thus, the principles of fluorochrome-based flow cytometry and the common processes that occur before and after flow cytometric assays are next briefly explained.

### **Basis of fluorescence-based flow cytometric assays of*Plasmodium falciparum*development**

The schematic diagram in Fig. 1 shows a flow cytometry method commonly used to assess *Plasmodium* development in culture (Fig. 1, left panel). A major change in asexual development is an increase in DNA synthesis and the number of nuclei. Thus, the use of nucleic acid-binding fluorescent chemicals is speculated to discriminate all asexual stages of *Plasmodium*. Flow cytometry is capable of analysing many thousands of cells on a single-cell basis in a short time. Each cell aligns in the flowing stream and is exposed to different wavelengths of light generated from the laser source (Fig. 1a, middle panel). Cell size and content can be measured using light scatter: forward scatter (FSC) and side scatter (SSC), respectively (green X-axis in the right panel of Fig. 1a). The use of FSCs and SSCs allows the exclusion of cell debris that is small; thus, quantifying a large number of cells is accurate, especially in the examination of parasitaemia post drug exposure. Upon excitation, the emitted fluorescence of nucleic acid-binding fluorochromes can be displayed based on their fluorescence intensities: high, intermediate and low (right panel of Fig. 1a) depending on the resolution of fluorescence intensity. Similar to the Giemsa-based microscopy data, the flow cytometer data indicate the proportion of *Plasmodium*-infected erythrocytes, known as parasitaemia. However, unlike microscopic examination, flow cytometry provides quantifiable data in a high-throughput, automatic manner, thus minimizing interassay variance. In drug susceptibility assays, a highly synchronized culture of *Plasmodium* parasites (laboratory-adapted strains or field-isolated strains) is first prepared and subsequently treated with various doses of drug. Mostly, synchronized cultures of ring-like *Plasmodium* parasites are incubated with drugs, and their growth is assessed mostly at the schizont stage. Hence, prior drug treatment and heterogeneity in *Plasmodium* parasites may confound the interpretation. Owing to the high resolution of the emitted fluorescence intensity, flow cytometry offers an option to determine the extent to which the culture is synchronous (Fig. 1b). After drug treatment, flow cytometry could provide information about not only parasitaemia but also the proportion of each developmental stage (Fig. 1c). The quantifiable data can be displayed as a dose-response curve. Moreover, a change in the proportion of each asexual stage provides the stage-specific effects of the tested compound or anti-malarial drug (Fig. 1d), allowing further investigation of a drug mechanism.

### **Applications of fluorochrome assays for the assessment of*Plasmodium*development**

Fluorochromes are classified into two categories of dyes: cell permeant and cell impermeant. Although the mechanism underlying fluorochrome transport across the cell membrane remains unknown, increased membrane transport of nucleosides, amino acids, and carbohydrates may account for cell permeability [39, 40].

#### Cell membrane-permeable fluorochromes

##### Hoechst

Hoechst dyes are bis-benzimides developed by Hoechst AG, and they have been used for DNA staining. Hoechst 33,258 and Hoechst 33,342 have similar excitation-emission spectra: they are excited by 350-nm ultraviolet light and emit blue-cyan fluorescent light around a maximum of 461 nm. By contrast, a 405-nm laser reportedly excited Hoechst 34,580, resulting in emission of light at the same wavelength as aforementioned above [41]. Hoechst dyes bind to all types of nucleotides located in a minor groove of double-stranded DNA (dsDNA); however, AT-rich DNA sequences preferentially interact with Hoechst dyes, resulting in an increase in fluorescence intensity [42]. Given the ability of the Hoechst dyes to permeate the cell membrane, they have been used for visualization of living cells in real time.

Despite such cell permeability, several reports demonstrated the utility of Hoechst 33,258 for *in vitro* drug susceptibility tests [34] and the detection of *P. falciparum* in human blood in a field study [35] using formaldehyde or guanidinium-HCl [43] as a fixative agent. Cell fixation and flow cytometric analysis were simultaneously performed in a single tube without a cell wash, simplifying the method. Moreover, cell fixation lysed non-parasitized erythrocytes, while *Plasmodium*-infected erythrocytes and leukocytes were still intact, allowing detection of seven parasites per thousand leukocytes [35]. Notably, the limit of detection could be higher due to the background fluorescent signal caused by artifact materials being a confounding factor in the accurate quantification of *P. falciparum*-infected erythrocytes. However, morphological analysis of formaldehyde-fixed parasitized cells at each intraerythrocytic stage remains difficult, limiting accurate identification of *Plasmodium* development. Therefore, cell fixation-free methods have been developed, allowing morphological observation and subsequent identification of developmental stages. A simpler single step of cell incubation with Hoechst 33,342, dihydroethidium and anti-CD45 at room temperature without washing or fixation enables identification of intraerythrocytic stages and seems suitable for high-throughput platforms [44]. Given that a UV laser is indispensable for detecting the fluorescence signals of Hoechst 33,342 and dihydroethidium and that only some flow cytometers are equipped with a UV laser, widespread use of the method is limited. Alternatively, a violet laser is also able to excite Hoechst 33,342 [45] and is commonly equipped with most flow cytometry; thus, the violet laser compensates for the lack of a UV laser.

##### Hydroethidine

Hydroethidine, also commonly known as dihydroethidium, has been used for detecting intracellular superoxide. Upon cell uptake, some reactive oxygen species oxidize hydroethidine in the cytoplasm. Ethidium, the oxidized form of hydroethidine, can enter the nucleus to intercalate into dsDNA. When the dye-DNA complex is excited by 535-nm light, ethidium emits fluorescence at 610 nm. Moreover, the ethidium-RNA complex could be excited by 370-nm light to emit 420-nm light, resulting in the detection of reticulocytes [44]. Given that most living cells metabolically produce reactive oxygen species, hydroethidine was first used to examine the viability and growth of *Babesia bovis*, an intraerythrocytic parasite of cattle [46]. For human malaria, hydroethidine revealed anti-malarial effects of leukocytes on the survival and development of *Plasmodium* parasites [47]. Its use in combination with the nucleic acid-binding dye thiazole orange yielded an increase in fluorescence intensity that correlated with the stage of *Plasmodium* development: multinuclear schizonts exhibited a fluorescence intensity higher than that of ring forms. Thus, the homogeneous intensity of fluorescence also allowed assessment of the synchronicity of *Plasmodium* parasites in a culture [48], an important factor for transcriptomic analysis. Importantly, hydroethidine was applicable for testing the anti-malarial drug susceptibility of asexual stages of field isolates [36] and cultured gametocytes [49] of *P. falciparum*. Nevertheless, based on morphological observations, nonviable parasites with fragmented DNA were regarded as viable ring-stage parasites after incubation with hydroethidine, warranting careful interpretation [50]. In a practical view, the requirement of a 37 °C incubation makes the use of hydroethidine relatively complicated compared to that of dyes that bind DNA at room temperature.

SYTO 61. SYTO 61 is a product in the SYTO® series commercialized by Thermo Fisher, and it binds to DNA and RNA. The DNA-SYTO-61 complex absorbs visible light maximally at 628 nm and emits red light at 645 nm. A single stain with SYTO 61 was able to discriminate between non-infected and infected erythrocytes, and to measure the development of *P. falciparum* via flow cytometry. Thus, the results from the anti-malarial chloroquine test with SYTO 61 were comparable to those of the [^3^H]-hypoxanthine incorporation test [51]. A combination of SYTO 61 with a fluorescent indicator of oxidative stress (dichlorofluorescin diacetate) was used to clearly demonstrate that the ring-form stage had the lowest level of 2′,7′‐dichlorofluorescein, whereas the trophozoite stage exhibited a higher level [51], which is in agreement with the increase in reactive oxygen species due to haemoglobin digestion. Although such a combination would support a screening of haemoglobin degradation inhibitors, exposure of dichlorofluorescin diacetate to strong light intensities results in its photochemical oxidation, confounding the interpretation of intracellular reactive oxygen species generation [52]. Hence, when aiming to elucidate oxidative stress using dichlorofluorescin diacetate, shortening the period of flow cytometric analysis is required.

##### Acridine orange

Acridine orange binds DNA and RNA via intercalation and electrostatic attraction between oppositely charged molecules. Acridine orange interacting with DNA can be maximally excited at 500 nm, whereas acridine orange bound to RNA is maximally excited by 460-nm light. As a result, the maximum emission of acridine orange occurs at 526 nm (green fluorescence) and 650 (red fluorescence) nm when the dye is bound to DNA or RNA, respectively, allowing discrimination between DNA and RNA. Despite cell permeability, acridine orange failed to detect *Plasmodium*-infected erythrocytes in a cell fixation-free setting [53], suggesting a requirement of permeabilization via chemicals or an increase in temperature. By contrast, Saito-Ito et al. [54] deployed a protocol in which parasitized erythrocytes were concurrently lysed using dodecyl methyl ammonium chloride, a lipid bilayer-dissociating disinfectant, and stained with acridine orange. This method allowed discrimination of *P. falciparum* parasites from lysed erythrocyte ghosts, white blood cells and platelets. By using a lysis-stain combination, the detection threshold of parasitaemia in a culture of *P. falciparum* and the dose of anti-malarial drug were similar to those obtained with a standard microscope [54]. To date, the number of reports in which acridine orange was used to analyse human malaria parasites without cell fixation or lysis is limited. Acridine orange permeated gametocytes of *P. falciparum* following more than 2 h of incubation at ambient temperature. This study demonstrated that acridine orange use did not affect gametocyte viability, allowing high-content confocal imaging of morphological transitions upon activation [55]. In rodent malaria models, *Plasmodium berghei* and *Plasmodium yoelii*-infected erythrocytes in peripheral blood could be detected using an acridine orange-based flow cytometry method. Owing to the detection of green and red fluorescence signals, *P. berghei* and *P. yoelii*-infected erythrocytes were discriminated from white blood cells [56]. Nevertheless, reticulocytes could confound the interpretation, especially in highly anaemic animals infected with *P. berghei* [57]. However, the low permeation by acridine orange is likely a drawback limiting its use for human malaria parasites.

##### Coriphosphine O

Coriphosphine O is a dsDNA intercalating and RNA loop-binding fluorochrome. In addition to its excitation by common 488-nm argon ion lasers, an advantage in using Coriphosphine O is its ability to emit light with two distinct spectra: green and orange fluorescence is emitted when it is bound to DNA and RNA, respectively. Moreover, given the large difference in the positions of the band maxima of the absorption and emission spectra (known as the Stokes shift) after it binds to nucleic acids, coriphosphine O is hence able to distinguish parasitized erythrocytes, especially those in the early ring-form stage, from non-infected cells at high resolution [58]. In a study of the host immune response against parasites, enumeration of parasite survival and death remains a challenge because nucleic acid-binding fluorophores are incapable of differentiating residual, fragmented DNA and RNA in dead cells from their respective intact forms in live cells. Thus, fluorochromes capable of binding to mitochondria in viable parasites have been employed [59]. A combination of MitoTracker, a mitochondria-staining fluorescent dye; Coriphosphine O; and a human CD45-specific antibody was able to enumerate live parasites and assess the effect of antibody-dependent cellular inhibition of leukocytes on *Plasmodium* growth *in vitro* [58]. The results obtained from this tricolour flow cytometry were comparable to those of Giemsa-based microscopy in the assessment of growth inhibition; however, this protocol is relatively complicated, requiring incubation at 37 °C and cell washing prior to the flow cytometric assay.

##### ViSafe Green

ViSafe Green (VSG) is an environmentally safe nucleic acid-binding fluorescent dye that has been applied for visualizing DNA and RNA in agarose and polyacrylamide gels. VSG can be activated by 250–300 nm (UV) light, and its emission exhibits a spectrum similar to that of ethidium bromide [60]. VSG is, therefore, an alternative to ethidium bromide for visualizing DNA and RNA in agarose gels. Recently, VSG was able to permeate *P. falciparum*-infected erythrocytes in all four major stages and that its fluorescence intensity depended on the intraerythrocytic stage of *P. falciparum* development. Of note, the VSG-based flow cytometric assay failed to distinguish gametocytes from schizonts [61]. The obtained relative values correlated well between the VSG assay and the gold standard microscopy method used to enumerate parasitized erythrocytes. Moreover, VSG-based flow cytometry was applicable for assessing the synchronicity of *P. falciparum* development in erythrocytes and the growth inhibitory effect of anti-malarial drugs *in vitro*. Compared to the use of other cell-permeant fluorochromes, the use of VSG is a relatively simple and fixation-free method that enumerates malaria-infected erythrocytes and assesses intraerythrocytic development in culture and in anti-malarial drug susceptibility assays. However, given DNA- and RNA-binding ability, the interpretation of VSG + cells is likely confounded by the presence of reticulocytes, nucleated erythrocytes and leukocytes. Moreover, VSG was not able to distinguish the early gametocyte stage in cultures containing schizonts. Thus, further validation and comparison with other fluorochromes are required to conclude its superiority.

#### Cell-impermeant fluorochromes

Some nucleic acid-binding fluorochromes require an additional step of cell membrane permeabilization. Aldehyde and ethanol are commonly used for fixation and permeabilization. Since both chemicals inevitably alter cell structure, morphological observation of malaria parasites is impossible after cell membrane permeabilization, a drawback of cell-impermeant fluorochromes.

##### Propidium iodide

Propidium iodide (PI) is an intercalating dye that binds to both DNA and RNA without nucleotide preference. Although unbound PI in solution can be excited, the emitted fluorescence signal is enhanced 20- to 30-fold after intercalation between nucleic acids. When bound to DNA, the excitation/emission maxima of PI are 535 nm (green)/617 nm (orange–red). PI has been widely applied for many purposes. First, since PI is unable to permeate the cell membrane, it can differentiate viable cells from nonviable cells that have lost membrane integrity. Second, the fluorescence intensity of PI is proportional to the DNA content, allowing cell cycle analysis. However, PI also binds to RNA; thus, it is necessary to eliminate RNA using RNase prior to staining with PI. Regarding applications of PI for malaria research, *Plasmodium* nucleic acids could be stained with PI after fixation with paraformaldehyde and glutaraldehyde [62]. In *in vitro* drug susceptibility tests of *P. falciparum*, the minimum concentration of drugs that inhibit *Plasmodium* parasite growth could be calculated based on a decrease in fluorescence intensity, and the results correlated with microscopy observations [63]. Moreover, owing to the high resolution of the emitted fluorescence intensity, the use of PI-based flow cytometry allowed the isolation of pure *P. falciparum* trophozoites from clinical blood samples for whole genome studies without host genome contamination [64].

##### SYBR Green I

SYBR Green I (SGI) is an asymmetrical cyanine dye (synthetic dyes belonging to the polymethine group) that was originally used for visualizing nucleic acids in gel electrophoresis, quantifying DNA in solution and detecting amplified products in quantitative real-time PCR. SGI interacts noncovalently with dsDNA via intercalation or minor groove binding [65]. Upon DNA binding, the DNA-SGI complex is capable of absorbing blue light (λ_max_ = 497 nm) and emitting green light (λ_max_ = 520 nm). SGI reportedly binds to single-stranded DNA and RNA, then emitting a fluorescent signal approximately 10-fold less intense than that for dsDNA binding [65, 66].

Regarding cell analysis, SGI was utilized for enumeration of viable cultured bacteria [67] and detection of viruses [68]. For *Plasmodium* parasites, there are two ways to use SGI-based flow cytometric analysis: with or without cell fixation. Izumiyama reported that without cell fixation, SGI staining of *P. falciparum*-infected erythrocytes was able to differentiate intraerythrocytic stages based on the fluorescence intensity at the wavelength of maximum intensity. Ring-form and young trophozoites exhibited the lowest fluorescence intensity, whereas mature schizonts had the highest fluorescence intensity. The developmental stages exhibiting an intermediate level of fluorescence intensity were the late trophozoite and young schizont stages [53]. The fixation-free protocol of SGI was also applicable for invasion assays; however, the requirement of a 37 °C incubation makes the protocol complicated and time-consuming [69]. In addition to its value in studying laboratory-adapted *P. falciparum* strains, fixation-free, SG-based flow cytometry was applicable to first round invasion-derived parasitaemia of clinical isolates [70]. For the cell fixation-based protocol, the use of paraformaldehyde as a fixative agent in the *P. falciparum* merozoite counting assay allowed the assessment of chloroquine effects on schizogony [53] and reinvasion [53, 69]. Moreover, a combination of erythrocyte lysis and 37 °C incubation reportedly increased resolution in the flow cytometric assay. Fluorescence intensity correlated with an increase in DNA content during 24-hour growth of synchronized *P. falciparum* [71]. However, without cell fixation, the resolution of the SGI fluorescence intensity was not well defined among the *Plasmodium* stages, limiting the assessment of parasite growth [44]. Therefore, the optimal use of SGI relies on methods to enhance cell permeability: cell fixation, cell lysis and increasing the temperature.

##### YOYO-1

YOYO-1, a cyanine dye, is a homodimer of oxazole yellow (known as YO) and binds to DNA via intercalation [72]. In solution, free YOYO-1 dye is activated by 458-nm light, resulting in maximum emission at 564 nm. When bound to dsDNA, YOYO-1 exhibits over a thousand-fold fluorescent enhancement. The DNA-intercalated YOYO-1 absorbs 489-nm light and maximally emits at 509 nm. The use of YOYO-1 was able to discriminate non-parasitized and parasitized erythrocytes in cultures of *P. falciparum*. Similar to other cell-impermeant dyes, fixation with formaldehyde is inevitable. Moreover, subsequent permeabilization with Triton X-100 (TX100) was deployed, ending 24 h before YOYO-1 staining [73]. The fluorescence intensity of YOYO-1 depended on developmental stage: multinucleated schizonts exhibited higher fluorescence intensity than ring-form schizonts. Owing to this behavior, YOYO-1-based flow cytometry served as a useful tool for screening biochemicals against *Plasmodium* development [73]. When this method was applied for the diagnosis of malaria in endemic areas, its sensitivity and specificity were comparable to those of standard microscopy; however, analyzing the fluorescence from reticulocytes was a drawback of the protocol because an appropriate negative control was required [74]. In both studies, RNA removal using RNase was included in the protocol. Overall, YOYO-1 is useful for *in vitro* culture of *Plasmodium* parasites but is not yet applicable for field isolates, largely due to its RNA-binding activity in reticulocytes.

##### PicoGreen

PicoGreen, an intercalating, cell-impermeant fluorochrome, specifically binds to dsDNA but not to ssDNA, RNA, or free nucleotides. The PicoGreen-dsDNA complex can be excited by 480-nm light and maximally emits at 520 nm. In contrast to its high fluorescence when bound to DNA, unbound PicoGreen emits fluorescence with a very low intensity. PicoGreen is resistant to photobleaching, which is an irreversible fading of fluorescence caused by light exposure; therefore, intra-assay variance is avoided owing to the high stability of the dye. Thus, it is suitable for high-throughput, multiwell-formatted assays and microplate readers. The use of PicoGreen in anti-malarial chloroquine assays gave a similar growth inhibitory concentration as the standard radioactive hypoxanthine uptake assay [75, 76]. Given the requirement of TX100 for cell permeabilization [75] or saponin for cell lysis [76], the use of PicoGreen is relatively complicated. Moreover, residual DNA of dead parasites and leukocyte contamination possibly confound the interpretation.

### Challenges and opportunities in the application of flow cytometry‐based assays

#### Viable parasite detection

A major drawback of the aforementioned DNA/RNA-staining fluorochromes is the inability to distinguish live and dead parasites owing to remnant DNA and/or RNA of *Plasmodium* inside the host cells. In most living eukaryotic cells, functioning mitochondria maintain their membrane polarization, whereas in metabolically inactive cells, nonviable cells lose such polarization [77]. Thus, living *Plasmodium* parasites could be detected based on mitochondrial function. Fluorescent dyes that bind to the polarized mitochondrial membrane, herein called mitochondrial dyes, are able to differentiate between living and dead cells [78]. MitoTracker CMXRos, a mitochondrial dye (8-(4′-chloromethyl) phenyl-2,3,5,6,11,12,14,15-octahydro-1 H,4 H,10 H,13 H-diquinolizino-8 H-xanthylium chloride), was used for this purpose. When applied with a combination of the growth inhibitory anti-malarial atovaquone and proguanil, the MitoTracker CMXRos was able to differentiate living parasite populations, which are characterized by intact mitochondrial membrane potential, from dead parasites whose mitochondrial membrane potential was compromised [59]. Although this method is useful to probe viable *Plasmodium* parasites, incubation at 37 °C is inevitable. Since nonviable cells have a compromised cell membrane, cell-impermeant fluorochromes are able to cross the compromised cell membrane and intracellularly bind to nucleic acids. Based on these properties, an approach to overcome this complicated protocol is to combine cell-permeant and cell-impermeant fluorochromes, especially those maximally emitting at different wavelengths. For instance, Hoechst 33,342, SYBR Green I or ViSafe Green can be combined with propidium iodide. Moreover, SYTOX® blue, a high-affinity nucleic acid dye that fluoresces, is able to enter only cells with compromised cell membranes. Thus, it has the potential to detect dead cells. To the best of my knowledge, there has been no report in which these combinations of fluorochromes have been deployed for *Plasmodium*-infected erythrocytes.

#### Anti-malarial drug sensitivity tests in the field

The emergence of anti-malarial drug resistance highlights the importance of drug susceptibility tests by which the existence and spread of resistant parasites can be detected and tracked. At present, given the availability of a compact, transportable flow cytometer, a combination of SYBR Green I and dihydroethidium, both cell permeant, has been applied for the detection of drug resistance in malaria endemic areas in Papua Province, Indonesia [36] and Thailand [37, 38].

**Fig. 2:**
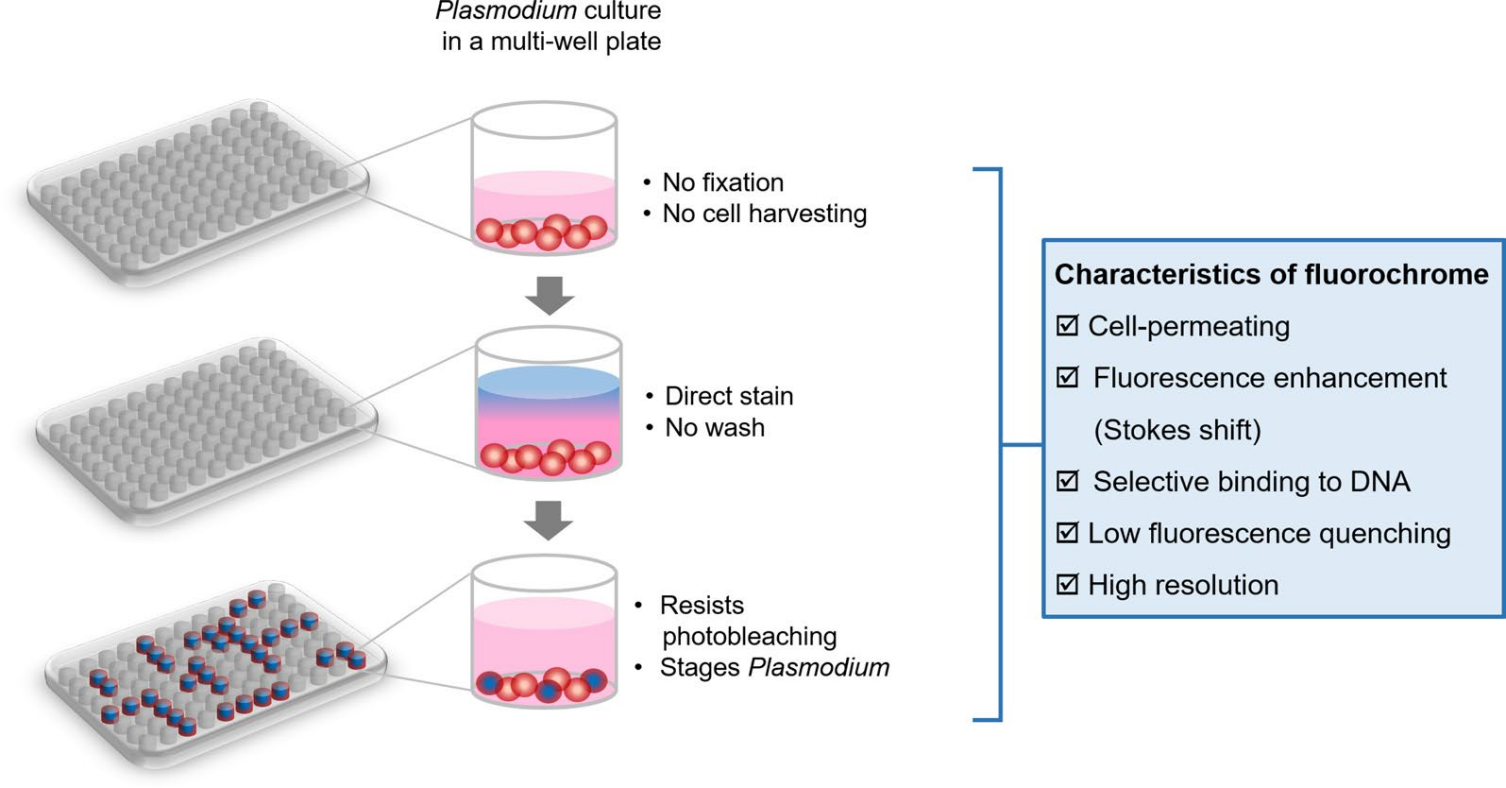
Proposed key features of fluorochrome-based flow cytometry for high-throughput antimalarial drug susceptibility testing. In a conventional drug susceptibility test, various doses of antimalarial drugs need to be evaluated; thus, the assay requires a protocol suitable for a multiwell format. Taking advantage of DNA-binding fluorochromes, key characteristics of flow cytometer-based drug susceptibility assays are proposed. Briefly, *Plasmodium*-infected erythrocytes are incubated with cell-permeant fluorochromes without cell fixation or harvesting. Given the fluorescence enhancement (increase in emitted fluorescence intensity or a Stokes shift upon binding to nucleic acids), the stained cells are directly exposed to laser without removal of unbound fluorochrome, eliminating a cell washing step. Highly selective DNA binding of fluorochromes minimizes the effects of confounding factors derived from RNA binding in reticulocytes. Since several wells are analysed, the fluorochrome must resist photobleaching. In addition, the high resolution of fluorescence intensity further allows discrimination of ring-form or mature trophozoites or schizonts, characterizing stage-specific drug resistance

Various doses of anti-malarial drugs need to be tested; thus, the assay required a protocol suitable for a multiwell operation. Here, key characteristics of fluorochromes are proposed for their use in flow cytometer-based drug susceptibility assays (Fig. 2). To allow anti-malarial drug susceptibility assays in a multiwell format, cultured *Plasmodium*-infected erythrocytes can be directly stained with a cell-permeant fluorochrome. Neither cell fixation nor harvesting are required. Given fluorescence enhancement (increasing fluorescence intensity or a Stokes shift upon binding to nucleic acids), the stained cells can be directly exposed to laser light without the removal of unbound fluorochrome. Thus, a washing step is not needed. Highly selective DNA binding of the fluorochrome will minimize the effects of confounding factors derived from RNA binding in reticulocytes. Since several wells are analysed, the fluorochrome must resist photobleaching. In addition, the high resolution of fluorescence intensity allows further discrimination of ring-form trophozoites, trophozoites and schizonts, characterizing stage-specific drug resistance.

However, there are two major hindrances in anti-malarial drug sensitivity tests. First, the use of nucleic acid-binding fluorochromes will lose accuracy owing to fluorescence signals of RNA in reticulocytes and remnant DNA (Howell-Jolly bodies) in some erythrocytes, both in erythrocytes or leukocytes in the peripheral blood of human individuals. To exclude such artifacts in blood, deploying fluorochrome binding specifically to RNA, antibody specific to transferrin receptor, highly expressed on immature enucleated erythrocytes and nucleated erythroid cells, or CD45, a common leukocyte antigen, allows more accurate identification of *Plasmodium*-infected erythrocytes [44, 58]. Second, the use of nucleic acid-binding fluorochromes is incapable of predicting whether drug-exposed parasites are alive or dead due to remnant DNA and/or RNA of *Plasmodium*. As discussed in viable parasite detection, there are two types of fluorescent dyes: one binds to the polarized mitochondrial membrane, and the other is a cell-impermeant dye capable of crossing a compromised cell membrane. Combinations of mitochondrial or cell-impermeant dyes with cell-permeant dyes will allow assessment of cidal effects in addition to growth inhibition.

#### Applications of the DRAQ5 fluorochrome

DRAQ5™ is a cell-permeable, DNA-binding anthraquinone. Given the lipophilicity of DRAQ5, it can cross phospholipid-enriched membranes without cell fixation [79]. Although DRAQ5 staining of zebrafish erythrocytes can be simply performed at ambient temperature [79], faster staining of *P. berghei* gametocytes with DRAQ5 was accomplished at a higher temperature [80]. Since DRAQ5 fluorescence intensity is correlated with DNA content and chromatin complexity, it could discriminate cell type based on the accessibility of DRAQ5 [81]. Moreover, its excitation and emission in the far red part of the spectrum allow the use of DRAQ5 with several fluorochromes. At present, there is no report of DRAQ5 being used for human malaria parasites. Thus, it will be interesting to see how DRAQ5 can be deployed for human malaria research.

#### Enhancement of erythrocyte membrane permeability to fluorochromes

Formaldehyde and glutaraldehyde partially solubilize lipids on the cell membrane [82], consequently increasing membrane permeability [83]. However, cell integrity is destroyed, leading to morphological changes in *Plasmodium*-infected erythrocytes [84] and basophilia of aldehyde-fixed biological samples [85]. TX100 is widely used for cell lysis in protein extraction methods. TX100 is a nonionic detergent composed of a hydrophilic polyethylene oxide chain and a hydrophobic aromatic hydrocarbon group. Given that it is a nonpolar, hydrophilic polyoxyethylene, TX100 is able to dissolve lipids from cell membranes, increasing cell permeability [86]. Following TX100 treatment, oval biconcave-shaped erythrocytes remain, while cell membrane permeability significantly increases, allowing intake of bovine serum albumin [87]. Recently, the use of the ionic detergent N-lauryl sarcosine increased the permeability of erythrocytes and enabled intracellular binding of antibodies for flow cytometric assays; however, a significant decrease in cell size was also observed [88]. Overall, fluorochrome permeation into the erythrocyte membrane could be enhanced using ionic or nonionic detergents such as Nonidet P-40.

### Perspectives

Despite many successful applications of fluorochrome-based flow cytometric assays in malaria research, none of them has become a widely-used method. High-cost and multistep processes, in contrast to the need for affordable and user-friendly tools, likely account for the restricted use of these assays in the malaria field. Recently, in attempts to develop low-cost, easy-to-use flow cytometers, a portable, compact flow cytometer and a microfluidic chip equipped with fluorescence readers have been invented [89, 90]. Thus, further exploration of the use of these devices is recommended. Moreover, a major drawback in the use of nucleic acid-staining fluorescent dyes in clinical samples is the inability to differentiate leukocytes and *Plasmodium*-infected erythrocytes. Owing to the larger amount of nuclear DNA in leukocytes, a possible solution is to search for fluorochromes exhibiting a wider range of fluorescence intensities (higher resolution); leukocytes and *Plasmodium*-infected erythrocytes emit fluorescence with highly different intensities. However, the invention of fluorochrome derivatives that are cell permeable, have high fluorescence enhancement, are selective for DNA binding and have fluorescence that quenches just slightly would further increase the use of flow cytometry in malaria research.

## Conclusions

The various uses of nucleic acid-binding fluorochromes and flow cytometry include enumeration of *Plasmodium*-infected erythrocytes and discrimination of *P. falciparum* growth in drug susceptibility assays. Studies deployed fluorochromes, which are capable and incapable of crossing the cell membrane to bind nucleic acids, for assessment of anti-malarial drug efficacy. Depending on purpose of study, the following guideline is proposed; (1) a combination of dihydroethidium with Hoechst 33,342 or thiazole orange is for drug susceptibility test, and discrimination of parasite maturation stage and viability, (2) a combination of SYTO61 with dichlorofluorescin is for assessment of oxidative stress, (3) a combination of Coriphosphine with MitoTracker is for discrimination of viable and nonviable *Plasmodium* parasites, and (4) a combination of dihydroethidium with thiazole orange is for study of transcriptiome. However, the previously reported hindrances, including the complexity of the process, inability to distinguish live and dead parasites, and low resolution and low quenching of fluorochromes, limit their widespread use in the aforementioned applications. To overcome these hindrances, the review highlights the key features to select and opportunities to use fluorochromes in flow cytometer-based drug susceptibility tests.

## Data Availability

Not applicable.

## Abbreviations

AT: Adenine and thymine
CD45: Cluster of Differentiation 45 (common leukocyte antigen)
DRAQ5: 1,5-bis{[2-(di-methylamino) ethyl]amino}-4,8-dihydroxyanthracene-9,10-dione
FSC: Forward scatter
HEPES: 4-(2-hydroxyethyl)-1-piperazineethanesulfonic acid
HCl: Hydrochloric acid
PI: Propidium iodide
RPMI 1640: Roswell Park Memorial Institute Medium 1640
SGI: SYBR Green I
SSC: Side scatter
TX100: Triton X-100
UV: Ultraviolet
VSG: ViSafe Green

## References

[1] Ashley EA, Pyae Phyo A, Woodrow CJ (2018). Malaria. Lancet.

[2] van der Pluijm RW, Imwong M, Chau NH, Hoa NT, Thuy-Nhien NT, Thanh NV (2019). Determinants of dihydroartemisinin-piperaquine treatment failure in *Plasmodium falciparum* malaria in Cambodia, Thailand, and Vietnam. A prospective clinical, pharmacological, and genetic study. Lancet Infect Dis.

[3] Rieckmann KH, Campbell GH, Sax LJ, Mrema JE (1978). Drug sensitivity of *Plasmodium falciparum*. An in vitro microtechnique. Lancet.

[4] Richards WH, Maples BK (1979). Studies on Plasmodium falciparum in continuous cultivation. I. The effect of chloroquine and pyrimethamine on parasite growth and viability. Ann Trop Med Parasitol.

[5] Nguyen-Dinh P, Trager W (1980). *Plasmodium falciparum* in vitro: determination of chloroquine sensitivity of three new strains by a modified 48-hour test. Am J Trop Med Hyg.

[6] Chotivanich K, Silamut K, Udomsangpetch R, Stepniewska KA, Pukrittayakamee S, Looareesuwan S (2001). Ex-vivo short-term culture and developmental assessment of Plasmodium vivax. Trans R Soc Trop Med Hyg.

[7] Bruce-Chwatt LJ (1981). Alphonse Laveran’s discovery 100 years ago and today’s global fight against malaria. J R Soc Med.

[8] Fleischer B (2004). 100 years ago: Giemsa’s solution for staining of plasmodia. Trop Med Int Health.

[9] Trager W, Jensen JB (1976). Human malaria parasites in continuous culture. Science.

[10] Silamut K, White NJ (1993). Relation of the stage of parasite development in the peripheral blood to prognosis in severe falciparum malaria. Trans R Soc Trop Med Hyg.

[11] Mathison BA, Pritt BS (2017). Update on malaria diagnostics and test utilization. J Clin Microbiol.

[12] Lambros C, Vanderberg JP (1979). Synchronization of *Plasmodium falciparum* erythrocytic stages in culture. J Parasitol.

[13] Lima RB, Rocha e Silva LF, Melo MR, Costa JS, Picanco NS, Lima ES (2015). In vitro and in vivo anti-malarial activity of plants from the Brazilian Amazon. Malar J.

[14] Dolabela MF, Povoa MM, Brandao GC, Rocha FD, Soares LF, de Paula RC (2015). Aspidosperma species as sources of anti-malarials: uleine is the major anti-malarial indole alkaloid from Aspidosperma parvifolium (Apocynaceae). Malar J.

[15] Srivastava K, Agarwal P, Soni A, Puri SK (2017). Correlation between in vitro and in vivo antimalarial activity of compounds using CQ-sensitive and CQ-resistant strains of *Plasmodium falciparum* and CQ-resistant strain of *P. yoelii*. Parasitol Res.

[16] Pathak M, Ojha H, Tiwari AK, Sharma D, Saini M, Kakkar R (2017). Design, synthesis and biological evaluation of antimalarial activity of new derivatives of 2,4,6-s-triazine. Chem Cent J.

[17] Saddala MS, Adi PJ (2018). Discovery of small molecules through pharmacophore modeling, docking and molecular dynamics simulation against *Plasmodium vivax* Vivapain-3 (VP-3). Heliyon.

[18] Singh J, Vijay S, Mansuri R, Rawal R, Kadian K, Sahoo GC (2019). Computational and experimental elucidation of *Plasmodium falciparum* phosphoethanolamine methyltransferase inhibitors: pivotal drug target. PLoS ONE.

[19] Desjardins RE, Canfield CJ, Haynes JD, Chulay JD (1979). Quantitative assessment of antimalarial activity in vitro by a semiautomated microdilution technique. Antimicrob Agents Chemother.

[20] Chulay JD, Haynes JD, Diggs CL (1983). *Plasmodium falciparum*: assessment of in vitro growth by [3H]hypoxanthine incorporation. Exp Parasitol.

[21] Niyibizi JB, Kirira PG, Kimani FT, Oyatsi F, Ng’ang’a JK (2020). Chemical synthesis, efficacy, and safety of antimalarial hybrid drug comprising of sarcosine and aniline pharmacophores as scaffolds. J Trop Med.

[22] Lingani M, Bonkian LN, Yerbanga I, Kazienga A, Valea I, Sorgho H (2020). In vivo/ex vivo efficacy of artemether-lumefantrine and artesunate-amodiaquine as first-line treatment for uncomplicated falciparum malaria in children: an open label randomized controlled trial in Burkina Faso. Malar J.

[23] Phong NC, Chavchich M, Quang HH, San NN, Birrell GW, Chuang I (2019). Susceptibility of *Plasmodium falciparum* to artemisinins and *Plasmodium vivax* to chloroquine in Phuoc Chien Commune, Ninh Thuan Province, south-central Vietnam. Malar J.

[24] Elabbadi N, Ancelin ML, Vial HJ (1992). Use of radioactive ethanolamine incorporation into phospholipids to assess in vitro antimalarial activity by the semiautomated microdilution technique. Antimicrob Agents Chemother.

[25] Lindert S, Tallorin L, Nguyen QG, Burkart MD, McCammon JA (2015). In silico screening for *Plasmodium falciparum* enoyl-ACP reductase inhibitors. J Comput Aided Mol Des.

[26] Makler MT, Hinrichs DJ (1993). Measurement of the lactate dehydrogenase activity of *Plasmodium falciparum* as an assessment of parasitemia. Am J Trop Med Hyg.

[27] Asahi H, Kanazawa T, Hirayama N, Kajihara Y (2005). Investigating serum factors promoting erythrocytic growth of *Plasmodium falciparum*. Exp Parasitol.

[28] Makler MT, Ries JM, Williams JA, Bancroft JE, Piper RC, Gibbins BL, Hinrichs DJ (1993). Parasite lactate dehydrogenase as an assay for *Plasmodium falciparum* drug sensitivity. Am J Trop Med Hyg.

[29] Noedl H, Wernsdorfer WH, Miller RS, Wongsrichanalai C (2002). Histidine-rich protein II: a novel approach to malaria drug sensitivity testing. Antimicrob Agents Chemother.

[30] Noedl H, Bronnert J, Yingyuen K, Attlmayr B, Kollaritsch H, Fukuda M (2005). Simple histidine-rich protein 2 double-site sandwich enzyme-linked immunosorbent assay for use in malaria drug sensitivity testing. Antimicrob Agents Chemother.

[31] Sinha S, Sarma P, Sehgal R, Medhi B (2017). Development in assay methods for in vitro antimalarial drug efficacy testing: a systematic review. Front Pharmacol.

[32] Noedl H, Wongsrichanalai C, Wernsdorfer WH (2003). Malaria drug-sensitivity testing: new assays, new perspectives. Trends Parasitol.

[33] Howard RJ, Battye FL, Mitchell GF (1979). *Plasmodium*-infected blood cells analyzed and sorted by flow fluorimetry with the deoxyribonucleic acid binding dye 33258 Hoechst. J Histochem Cytochem.

[34] van Vianen PH, Thaithong S, Reinders PP, van Engen A, van der Keur M, Tanke HJ (1990). Automated flow cytometric analysis of drug susceptibility of malaria parasites. Am J Trop Med Hyg.

[35] van Vianen PH, van Engen A, Thaithong S, van der Keur M, Tanke HJ, van der Kaay HJ (1993). Flow cytometric screening of blood samples for malaria parasites. Cytometry.

[36] Wirjanata G, Handayuni I, Prayoga P, Apriyanti D, Chalfein F, Sebayang BF (2015). Quantification of *Plasmodium ex vivo* drug susceptibility by flow cytometry. Malar J.

[37] Russell B, Malleret B, Suwanarusk R, Anthony C, Kanlaya S, Lau YL (2013). Field-based flow cytometry for ex vivo characterization of *Plasmodium vivax* and *P. falciparum* antimalarial sensitivity. Antimicrob Agents Chemother.

[38] Woodrow CJ, Wangsing C, Sriprawat K, Christensen PR, Nosten F, Renia L (2015). Comparison between flow cytometry, microscopy, and lactate dehydrogenase-based enzyme-linked immunosorbent assay for *Plasmodium falciparum* drug susceptibility testing under field conditions. J Clin Microbiol.

[39] Gero AM, Bugledich EM, Paterson AR, Jamieson GP (1988). Stage-specific alteration of nucleoside membrane permeability and nitrobenzylthioinosine insensitivity in *Plasmodium falciparum* infected erythrocytes. Mol Biochem Parasitol.

[40] Kirk K, Lehane AM (2014). Membrane transport in the malaria parasite and its host erythrocyte. Biochem J.

[41] Lelliott PM, Lampkin S, McMorran BJ, Foote SJ, Burgio G (2014). A flow cytometric assay to quantify invasion of red blood cells by rodent *Plasmodium* parasites in vivo. Malar J.

[42] Portugal J, Waring MJ (1988). Assignment of DNA binding sites for 4’,6-diamidine-2-phenylindole and bisbenzimide (Hoechst 33258). A comparative footprinting study. Biochim Biophys Acta.

[43] Smeijsters LJ, Zijlstra NM, Franssen FF, Overdulve JP (1996). Simple, fast, and accurate fluorometric method to determine drug susceptibility of *Plasmodium falciparum* in 24-well suspension cultures. Antimicrob Agents Chemother.

[44] Malleret B, Claser C, Ong AS, Suwanarusk R, Sriprawat K, Howland SW (2011). A rapid and robust tri-color flow cytometry assay for monitoring malaria parasite development. Sci Rep.

[45] Shapiro HM, Perlmutter NG (2001). Violet laser diodes as light sources for cytometry. Cytometry.

[46] Wyatt CR, Goff W, Davis WC (1991). A flow cytometric method for assessing viability of intraerythrocytic hemoparasites. J Immunol Methods.

[47] van der Heyde HC, Elloso MM, vande Waa J, Schell K, Weidanz WP (1995). Use of hydroethidine and flow cytometry to assess the effects of leukocytes on the malarial parasite *Plasmodium falciparum*. Clin Diagn Lab Immunol.

[48] Jouin H, Daher W, Khalife J, Ricard I, Puijalon OM, Capron M (2004). Double staining of *Plasmodium falciparum* nucleic acids with hydroethidine and thiazole orange for cell cycle stage analysis by flow cytometry. Cytometry A.

[49] Chevalley S, Coste A, Lopez A, Pipy B, Valentin A (2010). Flow cytometry for the evaluation of anti-plasmodial activity of drugs on *Plasmodium falciparum* gametocytes. Malar J.

[50] Engelbrecht D, Coetzer TL (2012). The walking dead: is hydroethidine a suitable viability dye for intraerythrocytic *Plasmodium falciparum*?. Parasitol Int.

[51] Fu Y, Tilley L, Kenny S, Klonis N (2010). Dual labeling with a far red probe permits analysis of growth and oxidative stress in *P. falciparum*-infected erythrocytes. Cytometry A.

[52] Halliwell B, Whiteman M (2004). Measuring reactive species and oxidative damage in vivo and in cell culture: how should you do it and what do the results mean?. Br J Pharmacol.

[53] Izumiyama S, Omura M, Takasaki T, Ohmae H, Asahi H (2009). *Plasmodium falciparum*: development and validation of a measure of intraerythrocytic growth using SYBR Green I in a flow cytometer. Exp Parasitol.

[54] Saito-Ito A, Akai Y, He S, Kimura M, Kawabata M (2001). A rapid, simple and sensitive flow cytometric system for detection of *Plasmodium falciparum*. Parasitol Int.

[55] Lucantoni L, Silvestrini F, Signore M, Siciliano G, Eldering M, Dechering KJ (2015). A simple and predictive phenotypic high content Imaging assay for *Plasmodium falciparum* mature gametocytes to identify malaria transmission blocking compounds. Sci Rep.

[56] Bhakdi SC, Sratongno P, Chimma P, Rungruang T, Chuncharunee A, Neumann HP (2007). Re-evaluating acridine orange for rapid flow cytometric enumeration of parasitemia in malaria-infected rodents. Cytometry A.

[57] Hein-Kristensen L, Wiese L, Kurtzhals JA, Staalsoe T (2009). In-depth validation of acridine orange staining for flow cytometric parasite and reticulocyte enumeration in an experimental model using *Plasmodium berghei*. Exp Parasitol.

[58] Tiendrebeogo RW, Adu B, Singh SK, Dodoo D, Dziegiel MH, Mordmuller B (2014). High-throughput tri-color flow cytometry technique to assess *Plasmodium falciparum* parasitaemia in bioassays. Malar J.

[59] Jogdand PS, Singh SK, Christiansen M, Dziegiel MH, Singh S, Theisen M (2012). Flow cytometric readout based on Mitotracker Red CMXRos staining of live asexual blood stage malarial parasites reliably assesses antibody dependent cellular inhibition. Malar J.

[60] Vivantis. https://www.vivantechnologies.com/index.php?option=com_content&view=article&id=1193:visafe-green-gel-stain&catid=83:nucleic-acid-dyes&Itemid=128.

[61] Kulkeaw K, Ketprasit N, Tungtrongchitr A, Palasuwan D (2020). A simple monochromatic flow cytometric assay for assessment of intraerythrocytic development of *Plasmodium falciparum*. Malar J.

[62] Pattanapanyasat K, Webster HK, Udomsangpetch R, Wanachiwanawin W, Yongvanitchit K (1992). Flow cytometric two-color staining technique for simultaneous determination of human erythrocyte membrane antigen and intracellular malarial DNA. Cytometry.

[63] Pattanapanyasat K, Thaithong S, Kyle DE, Udomsangpetch R, Yongvanitchit K, Hider RC (1997). Flow cytometric assessment of hydroxypyridinone iron chelators on in vitro growth of drug-resistant malaria. Cytometry.

[64] Boissiere A, Arnathau C, Duperray C, Berry L, Lachaud L, Renaud F (2012). Isolation of *Plasmodium falciparum* by flow-cytometry: implications for single-trophozoite genotyping and parasite DNA purification for whole-genome high-throughput sequencing of archival samples. Malar J.

[65] Zipper H, Brunner H, Bernhagen J, Vitzthum F (2004). Investigations on DNA intercalation and surface binding by SYBR Green I, its structure determination and methodological implications. Nucleic Acids Res.

[66] Vitzthum F, Geiger G, Bisswanger H, Brunner H, Bernhagen J (1999). A quantitative fluorescence-based microplate assay for the determination of double-stranded DNA using SYBR Green I and a standard ultraviolet transilluminator gel imaging system. Anal Biochem.

[67] Barbesti S, Citterio S, Labra M, Baroni MD, Neri MG, Sgorbati S (2000). Two and three-color fluorescence flow cytometric analysis of immunoidentified viable bacteria. Cytometry.

[68] Brussaard CP, Marie D, Bratbak G (2000). Flow cytometric detection of viruses. J Virol Methods.

[69] Theron M, Hesketh RL, Subramanian S, Rayner JC (2010). An adaptable two-color flow cytometric assay to quantitate the invasion of erythrocytes by *Plasmodium falciparum* parasites. Cytometry A.

[70] Bei AK, Desimone TM, Badiane AS, Ahouidi AD, Dieye T, Ndiaye D (2010). A flow cytometry-based assay for measuring invasion of red blood cells by *Plasmodium falciparum*. Am J Hematol.

[71] Karl S, Wong RP, St Pierre TG, Davis TM (2009). A comparative study of a flow-cytometry-based assessment of in vitro *Plasmodium falciparum* drug sensitivity. Malar J.

[72] Murade CU, Subramaniam V, Otto C, Bennink ML (2009). Interaction of oxazole yellow dyes with DNA studied with hybrid optical tweezers and fluorescence microscopy. Biophys J.

[73] Schuck DC, Ribeiro RY, Nery AA, Ulrich H, Garcia CR (2011). Flow cytometry as a tool for analyzing changes in *Plasmodium falciparum* cell cycle following treatment with indol compounds. Cytometry A.

[74] Campo JJ, Aponte JJ, Nhabomba AJ, Sacarlal J, Angulo-Barturen I, Jimenez-Diaz MB (2011). Feasibility of flow cytometry for measurements of *Plasmodium falciparum* parasite burden in studies in areas of malaria endemicity by use of bidimensional assessment of YOYO-1 and autofluorescence. J Clin Microbiol.

[75] Corbett Y, Herrera L, Gonzalez J, Cubilla L, Capson TL, Coley PD (2004). A novel DNA-based microfluorimetric method to evaluate antimalarial drug activity. Am J Trop Med Hyg.

[76] Quashie NB, de Koning HP, Ranford-Cartwright LC (2006). An improved and highly sensitive microfluorimetric method for assessing susceptibility of *Plasmodium falciparum* to antimalarial drugs in vitro. Malar J.

[77] Chen LB (1988). Mitochondrial membrane potential in living cells. Annu Rev Cell Biol.

[78] Chen LB (1989). Fluorescent labeling of mitochondria. Methods Cell Biol.

[79] Kulkeaw K, Inoue T, Ishitani T, Nakanishi Y, Zon LI, Sugiyama D (2018). Purification of zebrafish erythrocytes as a means of identifying a novel regulator of hematopoiesis. Br J Hematol.

[80] Billker O, Dechamps S, Tewari R, Wenig G, Franke-Fayard B, Brinkmann V (2004). Calcium and a calcium-dependent protein kinase regulate gamete formation and mosquito transmission in a malaria parasite. Cell.

[81] Smith PJ, Wiltshire M, Davies S, Patterson LH, Hoy T (1999). A novel cell permeant and far red-fluorescing DNA probe, DRAQ5, for blood cell discrimination by flow cytometry. J Immunol Methods.

[82] Doggenweiler CF, Zambrano F (1981). Extraction of phospholipids from aldehyde-fixed membranes. Arch Biol Med Exp (Santiago).

[83] Cheng R, Zhang F, Li M, Wo X, Su YW, Wang W (2019). Influence of fixation and permeabilization on the mass density of single cells: a surface plasmon resonance imaging study. Front Chem.

[84] Petithory JC, Ardoin F, Ash LR, Vandemeulebroucke E, Galeazzi G, Dufour M, Paugam A (1997). Microscopic diagnosis of blood parasites following a cytoconcentration technique. Am J Trop Med Hyg.

[85] Horobin RW (2011). How Romanowsky stains work and why they remain valuable - including a proposed universal Romanowsky staining mechanism and a rational troubleshooting scheme. Biotech Histochem.

[86] Koley D, Bard AJ (2010). Triton X-100 concentration effects on membrane permeability of a single HeLa cell by scanning electrochemical microscopy (SECM). Proc Natl Acad Sci USA.

[87] Xu C, Yang X, Fu X, Tian R, Jacobson O, Wang Z, Lu N, Liu Y, Fan W, Zhang F (2017). Converting red blood cells to efficient microreactors for blood detoxification. Adv Mater.

[88] Faure S, Van Agthoven A, Bernot D, Altie A, Grino M, Alessi MC, Malergue F, Canault M (2019). A novel rapid method of red blood cell and platelet permeabilization and staining for flow cytometry analysis. Cytometry B Clin Cytom.

[89] Joo S, Kim KH, Kim HC, Chung TD (2010). A portable microfluidic flow cytometer based on simultaneous detection of impedance and fluorescence. Biosens Bioelectron.

[90] Fan Y, Dong D, Li Q, Si H, Pei H, Li L, Tang B (2018). Fluorescent analysis of bioactive molecules in single cells based on microfluidic chips. Lab Chip.

[91] Dekel E, Rivkin A, Heidenreich M, Nadav Y, Ofir-Birin Y, Porat Z (2017). Identification and classification of the malaria parasite blood developmental stages, using imaging flow cytometry. Methods.

